# Supplementary material for: Egr2 induction in spiny projection neurons of the ventrolateral striatum contributes to cocaine place preference in mice
Source: eLife. 2021 Mar 16;10:e65228. doi: 10.7554/eLife.65228 (PMC8057818; doi:10.7554/eLife.65228)
Supplement: Transparent reporting form [file elife-65228-transrepform.docx]

***eLife’s* transparent reporting form**

We encourage authors to provide detailed information *within their submission* to facilitate the interpretation and replication of experiments. Authors can upload supporting documentation to indicate the use of appropriate reporting guidelines for health-related research (see [EQUATOR Network](http://www.equator-network.org/%20)), life science research (see the [BioSharing Information Resource](https://biosharing.org/)), or the [ARRIVE guidelines](http://www.plosbiology.org/article/info:doi/10.1371/journal.pbio.1000412) for reporting work involving animal research. Where applicable, authors should refer to any relevant reporting standards documents in this form.

If you have any questions, please consult our Journal Policies and/or contact us: [editorial@elifesciences.org](mailto:editorial@elifesciences.org).

**Sample-size estimation**

- You should state whether an appropriate sample size was computed when the study was being designed
- You should state the statistical method of sample size computation and any required assumptions
- If no explicit power analysis was used, you should describe how you decided what sample (replicate) size (number) to use

Please outline where this information can be found within the submission (e.g., sections or figure legends), or explain why this information doesn’t apply to your submission:

No explicit power analysis was performed. Sample sizes were determined based on prevailing standards, to ensure sufficient samples for statistical analysis in all experiments.

**Replicates**

- You should report how often each experiment was performed
- You should include a definition of biological versus technical replication
- The data obtained should be provided and sufficient information should be provided to indicate the number of independent biological and/or technical replicates
- If you encountered any outliers, you should describe how these were handled
- Criteria for exclusion/inclusion of data should be clearly stated
- High-throughput sequence data should be uploaded before submission, with a private link for reviewers provided (these are available from both GEO and ArrayExpress)

Please outline where this information can be found within the submission (e.g., sections or figure legends), or explain why this information doesn’t apply to your submission:

Replication:

Results included in the study are collected from multiple technical replicates obtained from independent experiments (biological replicates). Sample sizes are mentioned in the legend of each figure, and further consolidated in Supplementary files 6 and 7. Detailed description of sample identity, experimental origin, raw and processed data are available in the supplementary files submitted with the manuscript.

Data exclusion:

No data was removed during or after analysis of experimental results, other than removal of samples with <1 Million reads/sample from RNAseq experiments.

For baseline RNA-seq experiments, particularly, differential expression analysis was performed on a subset of samples to avoid batch effect, but the expression of the genes were plotted across all experimental replicates to confirm validity of the results. This information is mentioned in the methods section under

quantification and statistical analysis > analysis of shifts in baseline transcription

High-throughput sequence data

All raw sequencing data is available on NCBI GEO: GSE158588.

**Statistical reporting**

- Statistical analysis methods should be described and justified
- Raw data should be presented in figures whenever informative to do so (typically when N per group is less than 10)
- For each experiment, you should identify the statistical tests used, exact values of N, definitions of center, methods of multiple test correction, and dispersion and precision measures (e.g., mean, median, SD, SEM, confidence intervals; and, for the major substantive results, a measure of effect size (e.g., Pearson's r, Cohen's d)
- Report exact p-values wherever possible alongside the summary statistics and 95% confidence intervals. These should be reported for all key questions and not only when the p-value is less than 0.05.

Please outline where this information can be found within the submission (e.g., sections or figure legends), or explain why this information doesn’t apply to your submission:

Statistical analysis was performed with R version 3.4.4.

Data for all experiments in the study has been represented at the resolution of individual replicates. N numbers are stated in the figure legends and appropriate supplementary files. Description of statistics, p values and other relevant details are documented in **Supplementary file 6**, as well as in the legends and results sections.

(For large datasets, or papers with a very large number of statistical tests, you may upload a single table file with tests, Ns, etc., with reference to sections in the manuscript.)

**Group allocation**

- Indicate how samples were allocated into experimental groups (in the case of clinical studies, please specify allocation to treatment method); if randomization was used, please also state if restricted randomization was applied
- Indicate if masking was used during group allocation, data collection and/or data analysis

Please outline where this information can be found within the submission (e.g., sections or figure legends), or explain why this information doesn’t apply to your submission:

Animals were randomly assigned to experimental groups. Researchers were blinded about the experimental conditions during data acquisition and analysis whenever possible. Relevant information is detailed in the methods section of the manuscript.

**Additional data files (“source data”)**

- We encourage you to upload relevant additional data files, such as numerical data that are represented as a graph in a figure, or as a summary table
- Where provided, these should be in the most useful format, and they can be uploaded as “Source data” files linked to a main figure or table
- Include model definition files including the full list of parameters used
- Include code used for data analysis (e.g., R, MatLab)
- Avoid stating that data files are “available upon request”

Please indicate the figures or tables for which source data files have been provided:

Source data file for RNA-seq and smFISH experiments are available at NCBI GEO: GSE158588, and <http://dx.doi.org/10.17632/p5tsv2wpmg.1>, respectively.

Processed data files are included as Supplementary files, and are appropriately referenced in the text.
